# Pretreatment of different food rest materials for bioconversion into fungal lipid-rich biomass

**DOI:** 10.1007/s00449-018-1933-0

**Published:** 2018-04-13

**Authors:** D. Tzimorotas, N. K. Afseth, D. Lindberg, O. Kjørlaug, L. Axelsson, V. Shapaval

**Affiliations:** 1 Nofima AS, 1430 Ås, Norway; 2 Cambi Group AS, 1383 Asker, Norway; 3 The Faculty of Science and Technology, Norwegian University of Life Sciences, 1432 Ås, Norway

**Keywords:** Food rest materials, Pretreatment, Lipids, Fungi, Submerged fermentation

## Abstract

Food rest materials have the potential to be used as media components in various types of fermentations. Oleaginous filamentous fungi can utilize those components and generate a high-value lipid-rich biomass, which could be further used for animal and human use. One of the main limitations in this process is the pretreatment of food rest materials, needed to provide homogenization, sterilization and solubilization. In this study, two pretreatment processes—steam explosion and enzymatic hydrolysis—were evaluated for potato and animal protein-rich food rest materials. The pretreated food rest materials were used for the production of fungal lipid-rich biomass in submerged fermentation by the oleaginous fungus *Mucor circinelloides*. Cultivation media based on malt extract broth and glucose were used as controls of growth and lipid production, respectively. It was observed that media based on food rest materials can support growth and lipid production in *M. circinelloides* to a similar extent as the control media. More specifically, the use of potato hydrolysate combined with chicken auto-hydrolysate resulted in a higher fungal total biomass weight than using malt extract broth. When the same C/N ratio was used for glucose and rest materials-based media, similar lipid content was obtained or even higher using the latter media.

## Introduction

In the last decade, fungal biomass rich in lipids such as single cell oils (fungal SCOs), has emerged as a potential complementary lipid-rich raw material for food, feed and biodiesel production. Oleaginous fungi have the ability to accumulate up to 85% (w/w) lipid of their biomass as a storage compound [1, 2]. They accumulate lipids primarily in the form of triacylglycerols (TAGs), generally considered as storage lipids. The fatty acids profile of the TAGs varies between different sorts of fungal SCOs. In favorable cases this profile can be similar to vegetable and fish oil, rich in important polyunsaturated fatty acids (PUFA) such as, gamma-linolenic acid (GLA) [3]. This makes lipids from fungal SCOs perfectly qualified as ingredients in feed, food and pharmaceutical products.

A major bottleneck in the production of fungal biomass is the high raw material cost, which accounts for 70% of the production cost [4]. Utilization of cheap raw materials for the production of fungal lipid-rich biomass would reduce the production cost, and consequently the cost of the resulting lipid products [5, 6]. Among different raw materials available, food rest materials could be a good alternative for fungal SCOs production. These represent a wide range of raw materials which can replace both the carbon and nitrogen source in the fermentation medium. In addition, the lipid products could also potentially be accepted for human use, if all rest material handling and processing steps are performed according to respective laws and regulations.

Potatoes are one of the main carbohydrate source in the Western diet. The potato processing industries are producing various products such as chips, peeled potatoes and wedges, and during these processes they are currently generating a large amount of starch-based waste materials [7, 8]. This makes potato rest materials as one of the prospective low cost carbon sources available. Peptones or peptide extracts originating from fish, animal, milk, plant and bakers’ yeasts are traditional nitrogen sources for fungal fermentation. Production of peptones usually consists of a protein hydrolysis step (e.g., using enzymes or acid/base hydrolysis), followed by purification steps [9]. A replacement of expensive nitrogen sources with new ones based on protein-rich rest materials may contribute to a cost reduction in fungal lipid-rich biomass production. Animal-based food rest materials, such as bones with meat remnants or intestines, are rich in proteins and could be used as a nitrogen source for the production of the fungal biomass. To the authors’ knowledge there are no studies performed on the direct utilization of protein-rich animal rest materials in the fermentation for production of lipid-rich fungal biomass.

The efficient pretreatment of food rest materials, like potato and animal protein-rich rest materials, is a key factor for the successful bioconversion into fungal biomass. Among the different pretreatment procedures, steam explosion has been shown to be environmental and economical friendly, providing simultaneous homogenization and sterilization of materials [10, 11]. The main advantages of using steam explosion for the pretreatment of food rest materials are twofold: (1) high homogeneity of the pretreated materials that provided proper viscosity of the fermentation medium; and (2) high sterility of the pretreated materials that is necessary for carrying proper fermentation. In addition, depending on the biomass, high nutrient solubility can be achieved that provides a high bioconversion rate. The steam explosion process has been successfully used for the pretreatment of lignocellulose materials for biogas and bioethanol production [10, 11]. It has also been shown that steam explosion under progressive pressure increase conditions can increase starch solubility in potato materials. This leads to production of a low molecular weight liquid starch-rich material suitable for the bioconversion of it into bioethanol [12]. In addition to the steam explosion, enzymatic hydrolysis is a good alternative for the pretreatment of food rest materials. Despite of its high cost, it could be especially useful in cases where the complete solubilization of nutrients is not achieved or when other pretreatment methods, such as steam explosion, may generate a set of inhibitors.

Recently, Miniraj et al. reported the successful use of potato processing waste water for the production of fungal SCO by *Aspergillus* and *Mucor* fungi [13]. To the authors’ knowledge, there is no any study performed on evaluation of a steam explosion pretreatment and/or enzymatic hydrolysis of potato and animal protein-rich rest materials for bioconversion into fungal lipid-rich biomass. Thus, the main aim of this study is to study the effects of the pretreatment of potato and animal protein-rich food rest materials by steam explosion and enzymatic hydrolysis for the bioconversion into fungal lipid-rich biomass.

## Materials and methods

### Food rest materials

The following food rest materials were used in the study: (1) potato rest materials—sous-vide potatoes and potato peels were obtained from Bama AS, Norway. The potato and potato peel samples were freshly packed and stored at − 20 °C; (2) animal protein-rich rest materials—chicken rest materials (chicken intestines and chicken blood) and pork pulp (made from various animal parts and containing 25–30% beef and 70–75% pork) were obtained from Norilia AS, Norway. Chicken intestines, blood and pork pulp were received fresh and stored at − 20 °C.

### Experimental design

The evaluation of the pretreatment for selected food rest materials was performed in three consecutive steps. The first step was an initial evaluation of the soluble fraction resulting from steam explosion of food rest materials for production of fungal lipid-rich biomass. In this step, commercial carbon or/and nitrogen sources of the fermentation medium were replaced by the food rest material steam explosion liquid product. In the second step, further evaluation of the steam explosion pretreatment, as well as enzymatic hydrolysis was performed. The effect of different steam explosion conditions and use of enzymatic hydrolysis on (1) fungal growth and (2) lipid production was evaluated. The media based on the pretreated rest materials providing the best total biomass weight and lipid content were selected for the third step-validation, where different C/N ratios were used to study the production of fungal lipid-rich biomass.

### Pretreatment of food rest materials by steam explosion

Steam explosion is a method where steam is incorporated into a sample at high pressure, followed by a sudden decompression. The steam explosion unit (Cambi Group AS, Asker, Norway) used in the study has been described previously [14]. The electric steam boiler (Parat, Flekkefjord, Norway) provides steam to the 20 l. pressure vessel through an air-actuated valve, which maintains the vessel pressure at set point values. An air-actuated ball valve is regulating the explosion (rapid pressure drop) of the processed material to the flash tank. At the bottom of the tank, the processed material is collected within a removable bucket.

The pretreatment of food rest materials by steam explosion was performed in two runs and the weight of all of the materials was 3 kg in both runs. For the initial evaluation of steam explosion, the parameters used were 165 °C and 30-min exposure time. The steam exploded samples were centrifuged (5000*g*, at 4 °C for 10 min) and the soluble fraction was separated by centrifugation, lyophilized and stored at − 20 °C before the use. In case of animal protein-rich food rest materials, fat fraction was removed after the centrifugation and further the fat-free water-soluble fraction was lyophilized and stored at − 20 °C before the use. For the second step and third step of the evaluation and validation of pretreatment processes, different conditions for the steam explosion were tested: (1) for potato rest materials three different temperatures were used (135, 180 and 215 °C) with three exposure time points (10, 20 and 30 min) for each temperature, (2) for animal protein-rich rest materials one temperature (135 °C) with three exposure time points (10, 20 and 30 min) was used. The steam exploded samples were centrifuged (5000*g*, at 4 °C for 10 min) and soluble fraction was separated, lyophilized and stored at -20 °C before use.

### Hydrolysis of potato food rest materials

The potato rest materials were homogenized in a high-speed blender (Braun combimax700, Germany) and subsequently diluted 1:2 with water (w/w) to obtain a potato slurry. Enzymatic hydrolysis was performed using thermostable α-amylase from *B. licheniformis* (Sigma-Aldrich, Germany) with 5 KU enzyme activity at 80 °C for 3 h, followed by hydrolysis using a glucoamylase (Sigma-Aldrich, Germany) with an enzyme activity of 1.5 KU at 40 °C for 24 h. The enzymes were inactivated by either lowering the pH to pH = 2 (α-amylase), or boiling for 10 min (glucoamylase).

### Hydrolysis of animal protein-rich food rest materials

For the evaluation of enzymatic hydrolysis pretreatment only chicken rest materials were selected. The chicken food rest materials were homogenized by mincing and subsequently diluting two times with water (w/w). One part was boiled for 10 min to inactivate endogenous enzymes, and subsequently hydrolyzed at 60 °C for 3 h by Alcalase (1% w/w) (Novozyme, Denmark). The other part was hydrolyzed at 60 °C for 3 h by autolytic endogenous enzymes activated using acetic acid buffer at pH 2.5. In both cases, enzymes were inactivated by boiling samples for 10 min. Subsequently, the samples were centrifuged (5000*g*, 5 min) to separate the lipid phase and the peptide-rich water-phase. In enzymatic hydrolysis, centrifugation is considered a standard step for separating the lipid phase from the peptide-rich water-phase [15, 16].

### Elemental and chemical analysis of raw and pretreated food rest materials

Both raw and pretreated food rest materials were subjected to the elemental and chemical analysis. The elemental analysis of the food rest materials was done according to the method described previously by Ogner et al. [17]. The food rest materials were steam exploded and lyophilized prior to elemental analysis. Then the samples were burnt and their carbon and nitrogen composition was determined in percentages.

The chemical analysis was focused on determining protein, fat and carbohydrate content. The protein, fat, and carbohydrate are expressed in % of the media supernatant used in fermentations. The protein content of the samples was determined by ISO method 5983-2:2009. The fat content of the samples was determined by the Folch method including an acid hydrolysis step [18]. The starch and glucose content of the pretreated potato samples were determined using the total starch assay kit (Megazyme, Ireland) and a method according to McCleary et al. [19], respectively. During starch determination, thermostable α-amylase hydrolyses starch into maltodextrins and amyloglucosidase hydrolysis maltodextrins to d-glucose. Finally, hydrogen peroxide, which is released during oxidation of d-glucose to d-gluconate, was quantitatively measured in a colourimetric reaction with addition of peroxidase and production of quinone-imine dye.

### Media formulations based on pretreated food rest materials

#### Media for the initial evaluation

Two types of media were used for the initial evaluation (1) the type 1 media (M1–M5) containing predetermined percentages of the freeze dried soluble fraction resulting from steam exploded food rest materials alongside commercial medium components as carbon or nitrogen source; and (2) the type 2 media (M7–M10) containing only the lyophilized soluble fractions resulting from steam exploded food rest materials as carbon and nitrogen source (Table 1). Bacteriological peptone and malt extract were used as commercial medium components (Oxoid, England), and malt extract broth (M6) was used as a growth control medium. All types of media used were autoclaved at 121 °C for 15 min prior to fermentation.

**Table 1:** Media formulated for the initial evaluation of the steam exploded food rest materials

Media no.	Media name	Carbon source	Nitrogen source
M1	Potato-based	12 g potato	0.6 g peptone
M2	Potato peel-based	12 g potato peel	0.6 g peptone
M3	Chicken intestines-based	4 g malt extract	3 g chicken intestine
M4	Pork pulp-based	4 g malt extract	3 g pork pulp
M5	Blood-based	4 g malt extract	3 g chicken blood
M6	Malt extract broth	4 g malt extract	0.6 g peptone
M7/M8	Potato-chicken intestine	3 g/ 6 g potato	1 g chicken intestine
M9/M10	Potato peel-chicken intestine	3 g/ 6 g potato peel	1 g chicken intestine

The composition of the media described below is per 200 ml used in the fermentation

#### Media for the pretreatment evaluation

The media for the pretreatment evaluation were based on differently pretreated food rest materials combined with commercial medium components as carbon and nitrogen source. After the pretreatment, food rest materials were centrifuged (5000*g* for 10 min) and water-soluble fat free fractions (WSFFs) were separated and lyophilized. Two pretreatment processes were used—steam explosion and hydrolysis. Two types of media, depending on the type of rest materials, were formulated (Table 2). The type 3 media (M11–M20) were based on the potato and potato peel materials processed by hydrolysis (M11) and steam explosion (M12–M20). The type 4 media (M21–M25) were based on the chicken rest materials processed by the hydrolysis (autolytic and enzymatic) (M21–M22) and steam explosion (M23–M25) (Table 2). Bacteriological peptone and malt extract used were from Oxoid, England. All types of media used were autoclaved at 121 °C for 15 min prior to fermentation.

**Table 2:** Media formulated for the evaluation of steam explosion and enzymatic hydrolysis

Media no	Media name	Carbon source	Nitrogen source
M11	Potato hydrolysate-based	15 ml potato hydrolysate supernatant	0.6 g peptone
M12	SE Potato-based	15 ml potato WSFF SE: 135 °C, 10 min	
M13		15 ml potato WSFF SE: 135 °C, 20 min	
M14		15 ml potato WSFF SE: 135 °C, 30 min	
M15		15 ml potato WSFF SE: 180 °C, 10 min	
M16		15 ml potato WSFF SE: 180 °C, 20 min	
M17		15 ml potato WSFF SE: 180 °C, 30 min	
M18		15 ml potato WSFF SE 215 °C, 10 min	
M19		15 ml potato WSFF SE 215 °C, 20 min	
M20		15 ml potato WSFF SE 215 °C, 30 min	
M21	Chicken autolytic hydrolysate	4 g malt extract	10 ml fat-free chicken intestine (auto-hydrolysate) WSFF
M22	Chicken enzymatic hydrolysate		10 ml fat-free chicken intestine (alcalase) WSFF
M23	SE chicken-based		10 ml WSFF chicken intestine SE: 135 °C, 10 min
M24			10 ml WSFF chicken intestine SE: 135 °C, 20 min
M25			10 ml WSFF chicken intestine SE: 135 °C, 30 min

The composition of the media described below is per 200 ml used in the fermentation

*SE* steam explosion, *WSFF* water soluble fat free fraction

#### Media used for the validation

In the third step, for the final screening two groups of media were used, both based on potato hydrolysate. These were combined with different amounts of yeast extract or the fat-free chicken intestines auto-hydrolysate (Table 3). The glucose-based media were used as lipid production control media, using the same C/N ratios as the two groups of media. All types of media used were autoclaved at 121 °C for 15 min prior to fermentation. The composition of the media described in Table 3, is per 100 ml used in the fermentation.

**Table 3:** Media for the validation, where enzymatically hydrolyzed potato was combined with either yeast extraction or WSFFs of chicken intestines auto-hydrolysate at C/N ratios 8 and 20

Media no.	Media name	Carbon source	Nitrogen source	C/N ratio
M26	Potato hydrolysate-based	100 ml enzymatically hydrolyzed potato WSFF	1.32 g of yeast extract	20
M27			3.3 g of yeast extract	8
M28			10 ml of WSFF of chicken intestines auto-hydrolysate	20
M29			25 ml of WSFF of chicken intestines auto-hydrolysate	8
M30	Glucose-based	6.6 g of glucose	1.32 g of yeast extract	20
M31		6.6 g of glucose	3.3 g of yeast extract	8
M32		8 g of glucose	0.3 g of yeast extract	106

### Fungal strain and growth conditions

The oleaginous fungus *Mucor circinelloides* VI04473 (Norwegian Veterinary Institute, Oslo, Norway) was used in the study. For the spore inoculum preparation, *M. circinelloides* VI04473 was cultivated on malt extract agar (MEA) (Sigma-Aldrich, Germany) for 5 days at 25 °C. The spore inoculum was prepared by adding 5 ml solution of 8.5 g l^−1^ NaCl containing 0.1% Tween 80 to the MEA plates (Sigma-Aldrich, Germany). The spores were collected and stored at 4 °C. For every experimental step, a fresh spore suspension was prepared. The fungal fermentations were performed in 500 ml baffled shaking flasks (Duran, Germany) with cotton plugs, where 200 µl of spore inoculum was added to 200 ml of growth medium. The flasks were incubated in shaker Innova 4000 (New Brunswick Scientific, Germany) during 4 days (28 °C, 180 rpm). After fermentation, biomass was collected and washed by filtration (Whatman grade 1 filter paper) and lyophilized using an Alpha 1–2 LD plus freeze-drier (Martin Christ, Germany) (− 52 °C, 0.008 mbar). The lyophilized biomass was stored at − 20 °C until analysis. The fermentations for initial evaluation of steam explosion and hydrolysis evaluation were performed with one replicate, while fermentations for the validation were performed with three replicates.

### Direct FAME esterification procedure

The direct fatty acid methyl ester (FAME) esterification was performed according to O’Fallon et al. [20] with some modifications: 0.1 g of lyophilized fungal biomass was dissolved in 5.3 ml of methanol (MeOH). Further, mycelium was grinded using mortar and pestle, then transferred in a glass test tube (Duran, Germany) with sealed cap and sonicated for 30 s with a single-tip sonicator (Sonics & Materials, USA). After sonication, the tube was placed on ice and 0.7 ml of 10 N KOH (dissolved in distilled water) and 1 ml of internal standard (C13:0, 1 mg ml^−1^ dissolved in MeOH) was added. The solution was mixed and tubes were incubated (55 °C, 1.5 h) with an end-over-end mixing regime every 20 min during incubation. After incubation, tubes were cooled in a water bath, thereafter 0.58 ml of 24 N H_2_SO_4_ (prepared in distilled water) was added. This solution was mixed by vortex for ~ 5 min. Further, tubes were incubated (55 °C, 1.5 h) with end-over-end mixing regime every 20 min during incubation. After incubation, tubes were cooled in a water bath, and 2 ml of hexane was added. The solution was mixed by vortex for ~ 5 min. The top hexane layer containing FAMEs was separated by low-speed centrifugation (800 g). To remove any remaining water, 0.05 g per vial of anhydrous Na_2_SO_4_ was added.

### Gas chromatography analysis

FAME composition was determined by gas chromatography (HP6890N, Agilent Technologies) with a split injector, auto-sampler (7683B) and flame ionization detection, using a SGE BPX70 column (60 m, 0.25 mm ID 0.25 µm). Helium was used as carrier gas (1.4 ml min^−1^). Oven temperature was initially set at 70 °C, 1 min, followed by an increase of 30 °C per min up to 170 °C. Thereafter, a 1.5 °C increase per min up to 200 °C, and finally 3 °C increase per min up to 220 °C with a 5 min holding time. Individual fatty acids were identified using external standard FAME mixture (Supelco, USA). The quantification of lipids (C_n_) was based on the internal standard calibration using response factors (RF) and the internal standard (C13:0) areas and concentrations:

$${C_n}=~{\text{Concentration~~}}{C_{13:0~}} \times ~\frac{{{\text{Area~}}{{\text{C}}_n}~ \times {\text{RF}}~{C_{13:0}}}}{{{\text{Area~}}~{C_{13:0~}} \times {\text{RF}}~{C_n}}}~{\text{and~R}}{{\text{F}}_n}=~\frac{{{\text{Area}}~n~}}{{{\text{Concentration}}~n~}}.$$

Fatty acid concentrations are expressed in percentages of the sum of saturated fatty acids (SAT), monounsaturated fatty acids (MUFA) and polyunsaturated fatty acids (PUFA).

## Results

### Initial screening of food rest materials for fungal lipid-rich biomass production

The aim of the initial screening was to evaluate the potential of using steam explosion for the pretreatment of food rest materials and if it is possible to perform further bioconversion of the resulting soluble fractions into fungal lipid-rich biomass by oleaginous fungus *M. circinelloides*. To evaluate the nutrition value and to achieve a correct C/N ratio in the final validation of the rest materials-based media, the elemental and chemical analysis of steam exploded potato and protein-rich animal rest materials was performed (Table 4). It was observed that potato and potato peel have the highest C/N ratio, while the lowest C/N ratio was observed for blood (Table 4). The protein content of blood was the highest followed by chicken intestines and pork pulp (Table 4). In addition, chicken rest materials and pork pulp showed high content of fat (Table 4). The carbohydrate content of potato and potato peel was the highest among all the tested food rest materials.

**Table 4:** The elemental and chemical analysis of steam exploded and, subsequently, lyophilized food rest materials

Material	Elemental analysis			Chemical analysis			
	C%	N%	C/N	Protein %	Fat %	Carbohydrate %	Ash %
Potato	43.62	0.96	45.28	5.7	1.6	83.4	2.2
Potato peel	43.69	0.86	50.78	4.8	n.a	89.2	2.2
Chicken intestines	61.8	7.47	8.27	42.4	46.1	3.5	3.4
Pork pulp	59.25	7.13	8.31	42.4	47.1	0.5	8.2
Blood	49.78	14.56	3.42	85.2	7.2	n.a	6.6

**Fig. 1:**
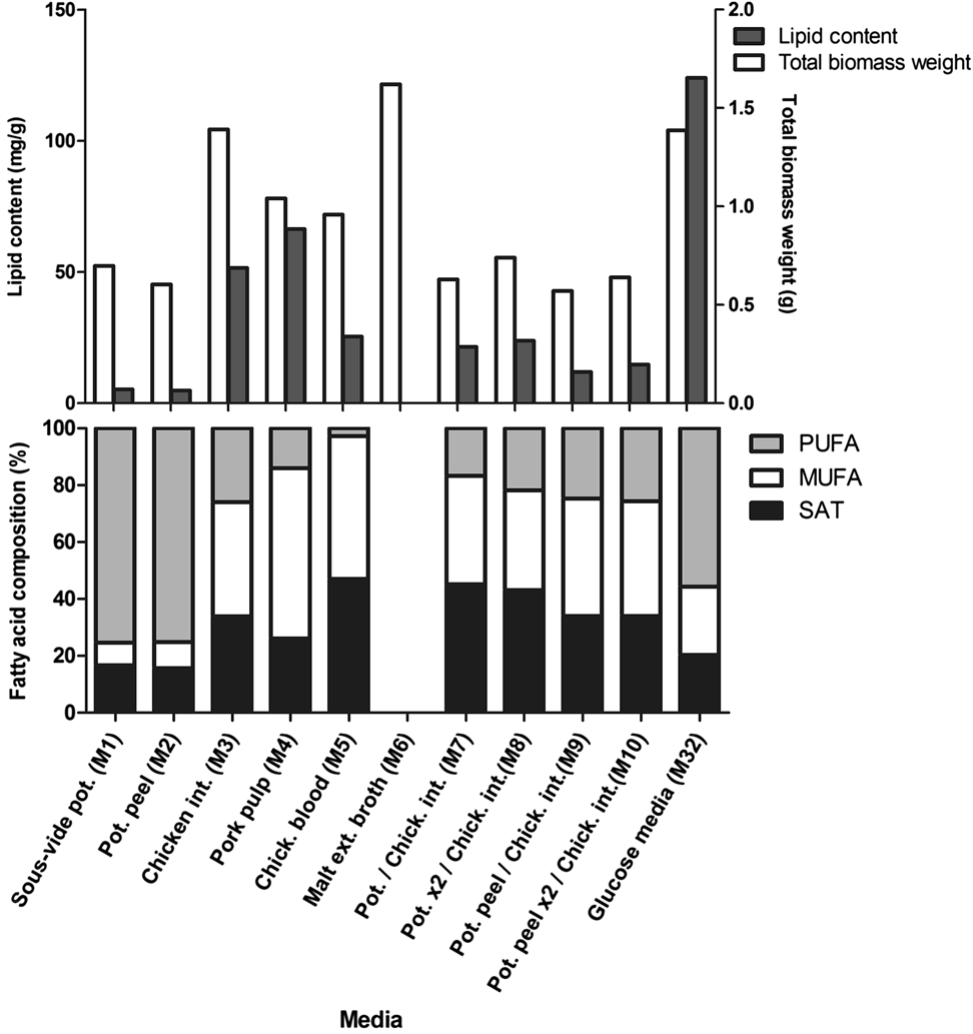
Total biomass weight, total lipid content, and fatty acid profile of *M. circinelloides* grown on initial screening media (M1–M5, and M7–M10) and control growth and lipid production media (M6 and M32). The total biomass weight is expressed in g, the total lipid content in mg/g of biomass and the fatty acid profiles (*SAT* saturated fatty acids, *MUFA* monounsaturated fatty acids, *PUFA* polyunsaturated fatty acids) in percentages (%) of the total lipid content

The initial screening was performed, first, for media based on food rest materials combined with commercial media components (M1–M5), then the most promising types of food rest materials were used for the screening of media based on rest materials without commercial components added (M7–M10). The total biomass weight, total lipid content and fatty acid profiles are provided in Fig. 1. The results showed that media based on food rest materials combined with commercial media components (M1–M5) supported fungal growth well, while the total biomass weight was lower than with the control growth medium (M6) (Fig. 1). The medium based on chicken intestines (M3) resulted in the highest total biomass weight, while potato (M1) and potato peel-based media (M2) provided the lowest amount of biomass compared to other tested materials. The total lipid content in the biomass obtained from these media was lower than on control lipid production medium (M32) (Fig. 1). The highest total lipid content was observed for the medium based on pork pulp (M4) followed by chicken intestines-based medium (M3), while the lowest total lipid content was observed for potato (M1) and potato peel-based medium (M2) (Fig. 1). The biomass obtained after cultivation on potato (M1) and potato peel-based media (M2) contained the highest percentage of PUFA. The fatty acid profile of biomass obtained after growth on chicken intestines-based medium (M3) showed higher amount of PUFA than on pork pulp-based medium (M4), and MUFA were the dominating fatty acids in the biomass obtained from both media.

To summarize based on the above presented results, potato and potato peel are the rest materials providing the highest percent of PUFA in the fungal biomass while chicken intestines providing the highest total biomass weight and also showing a relatively high total lipid content.

In the second step of the initial screening we evaluated media based on food rest materials—potato, potato peel, and chicken intestines without commercial media components added (M7–M10). It was observed that the total biomass weight and total lipid content for the media based only on the food rest materials (M7–M10) was much lower than for the media where rest materials combined with commercial media components (M1–M5) and control media (M6 and M32). The total biomass weight and total lipid content for the media where peeled potato was combined with chicken intestines (M7 and M8) was higher than for the media where potato peel was combined with chicken intestines (M9 and M10) (Fig. 1). The fatty acid profile of lipids extracted from the biomass grown on the media with potato peel (M9 and M10) showed slightly higher amount of polyunsaturated fatty acids (PUFAs) than on the media with peeled potato (M7 and M8). Concomitantly, the amount of saturated fatty acids (SAT) was lower on the potato peel medium than on medium with peeled potato (Fig. 1). The total biomass weight, total lipid content, and amounts of PUFA was lower for media based on the rest materials than for the control media (M6 and M32).

Because the potato-based media (M7–M8) resulted in a higher fungal total biomass weight and higher total lipid content compared to potato peel, it was selected as a suitable carbon source to replace malt extract or glucose in the growth and lipid production media. To replace the nitrogen source in the growth and lipid production media, chicken intestines were selected.

### Evaluation of the pre-processing procedure for potato and chicken rest materials

**Fig. 2:**
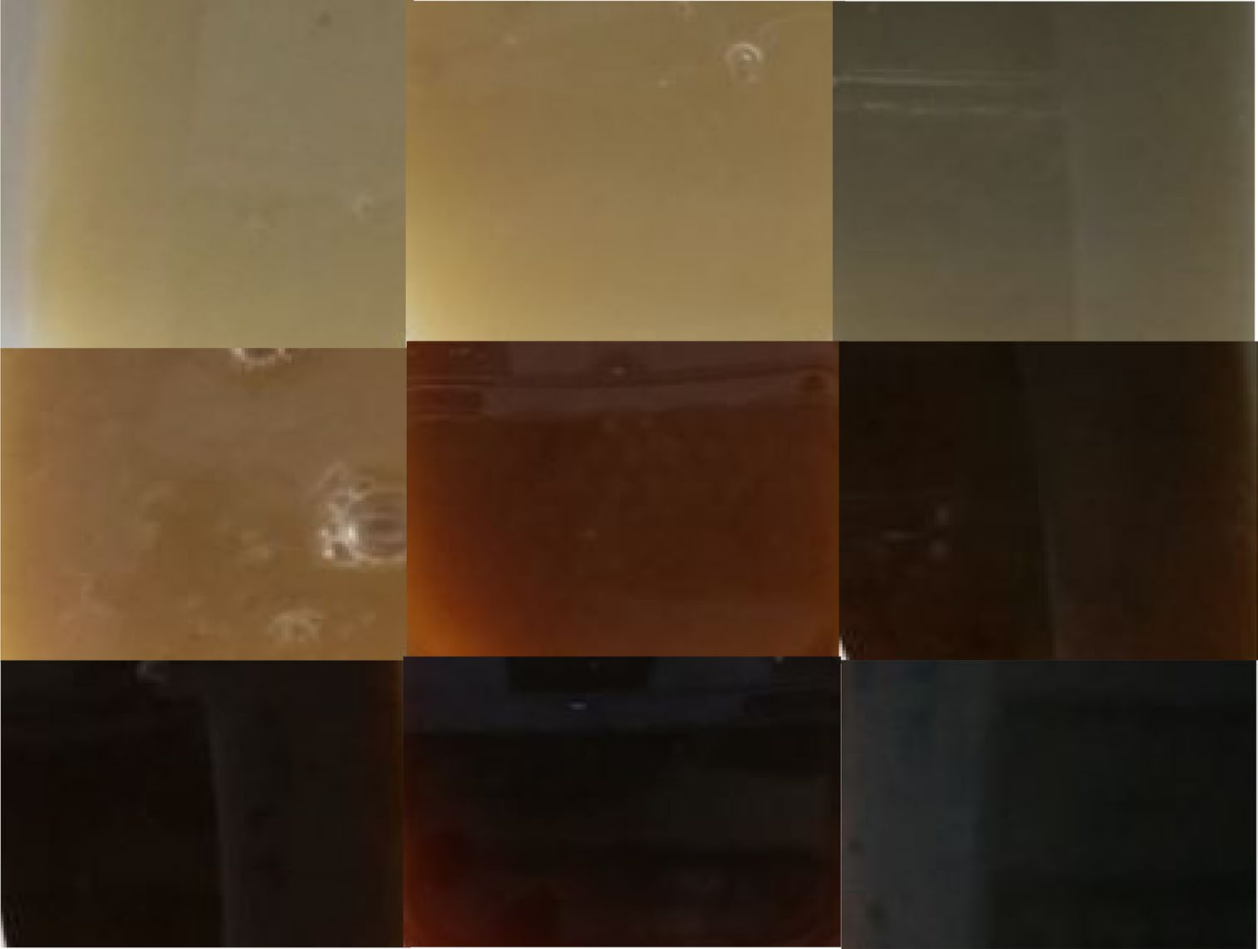
The color change of steam exploded potato, using various steam explosion condition. The boxes on the top represent the potato samples with steam explosion at the lowest temperature (135 °C), the boxes in the middle are samples from medium temperature treatment (180 °C), and the boxes at the bottom represent samples from the highest temperature (215 °C). From left to right on all rows, samples treated at the stated temperature for 10, 20 and 30 min, respectively

To increase the availability of nutrients, such as carbon from potato and nitrogen from chicken rest materials, the evaluation of pre-processing procedure was performed. The pre-processing was performed by steam explosion and hydrolysis (enzymatic and autolytic). Steam explosion was applied to potato and chicken intestine rest materials, and the main parameters evaluated were temperature and exposure time. The enzymatic hydrolysis was performed on potato rest materials and on chicken intestine rest materials. For the chicken intestine rest materials we also applied autolytic hydrolysis, utilizing the endogenous enzymes in the digestive system. The use of different temperatures and exposure time in steam explosion of potato rest materials did not result in significant changes in the amount of soluble starch and glucose released. One exception was at 215 °C and an exposure time of 20 and 30 min, where the amount of soluble starch and glucose as well as total amount of carbohydrates released was the highest (Table 5). However, the use of very high steam explosion temperatures may cause considerable glucose losses due to caramelization, as it was observed in our study (Fig. 2). From these results it becomes clear that utilization of steam explosion as a pre-processing method for potato rest materials is not a very effective approach. This is evident by the small proportion of carbohydrates (18%) solubilized during processing at various temperature and time conditions (Table 5).

**Table 5:** Results from the chemical analysis of potato and chicken intestines rest materials and pre-processed (steam exploded or enzymatically treated) potato and chicken intestines, used for the preparation of media (M11–M25)

Sample (medium #)	Steam explosion conditions °C/min	Protein %	Fat %	Ash %	Carb. %	Glucose (mg/100 ml)	Starch (mg /100 ml)
Potato	n.a.	1.4	0.3	0.7	18.9	n.a.	n.a.
M11	n.a.	0.4	n.a.	0.3	5.6	4766	0.53
M12	135/10	0.3	n.a.	0.2	0.7	130	0.27
M13	135/20	0.3	n.a.	0.2	1	82	0.30
M14	135/30	0.1	n.a.	0.1	0.4	38	0.29
M15	180/10	0.3	n.a.	0.2	0.8	98	0.34
M16	180/20	0.2	n.a.	0.1	0.5	24	0.31
M17	180/30	0.2	n.a.	0.1	0.7	17	0.34
M18	215/10	0.2	n.a.	0.1	2.2	24	0.82
M19	215/20	0.2	n.a.	0.1	1.8	137	1.39
M20	215/30	0.1	n.a.	0.1	2	239	1.60
Chicken intestines	n.a.	12.7	16.6	1	0.6	n.a.	n.a.
M21	n.a.	5.6	0.3	0.4	1.2	n.a.	n.a.
M22	n.a.	5.5	0.1	0.4	1.3	n.a.	n.a.
M23	135/10	1.1	0.8	0.2	0.6	n.a.	n.a.
M24	135/20	1.4	1	0.2	0.4	n.a.	n.a.
M25	135/30	1	0.5	0.1	0.5	n.a.	n.a.

The amount of glucose and starch is expressed in mg per 100 ml of the soluble supernatant used in media preparation (n.a.: not available)

**Fig. 3:**
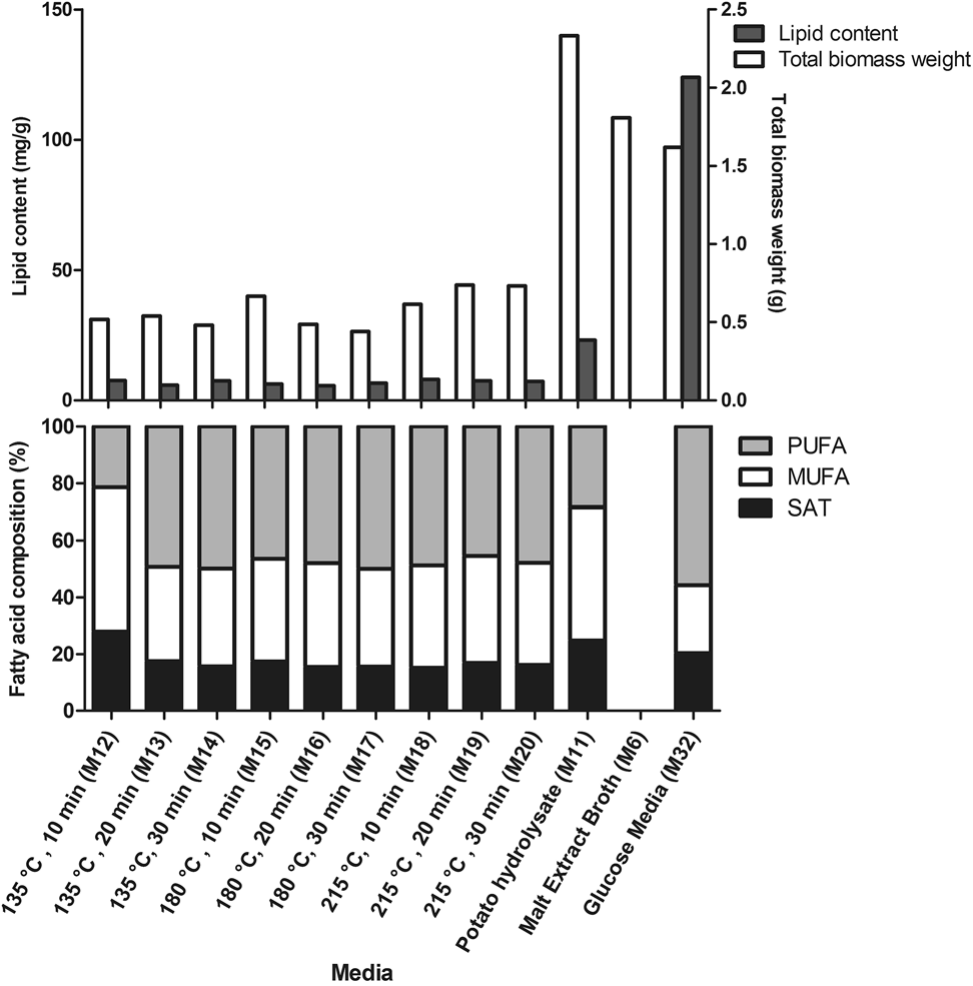
The total biomass weight, total lipid content and fatty acid profile of *M. circinelloides* grown on differently pre-processed potato-based media (M11–M20). The total biomass weight is expressed in g, the total lipid content in mg/g of biomass, and the fatty acid profiles (SAT, MUFA and PUFA) in percentages (%) of the total lipid content

The pre-processed potato and chicken intestine rest materials were combined with the commercial media components: pre-processed potato rest materials were combined with peptone (M11–M20), and pre-processed chicken intestine rest materials were combined with malt extract (M21–M25). All formulated media were tested for the fungal growth and lipid production. When these media (M11–M20) were used for fungal growth and lipid production, it was observed that all of them supported the growth of fungi and lipid production. Some variations were seen, dependent on what type of pre-processing has been used for the rest materials (Fig. 3). The medium based on the hydrolyzed potato (M11) showed the highest total biomass weight compared to all other rest materials-based media and the growth control medium (M6). The lipid production in fungi grown on the hydrolyzed potato medium (M11) was the highest compared to other rest materials-based media, but it was still very low in comparison to the lipid production control medium (M32) (Fig. 3). While amount of PUFA in the fungi grown on the hydrolyzed potato was lower than on media based on the steam exploded potato, and amount of MUFA and SAT was higher. Another interesting observation was related to the fact that various steam explosion conditions used for processing potato did not have a strong effect on the total biomass weight and lipid content, as well as the lipid profile.

**Fig. 4:**
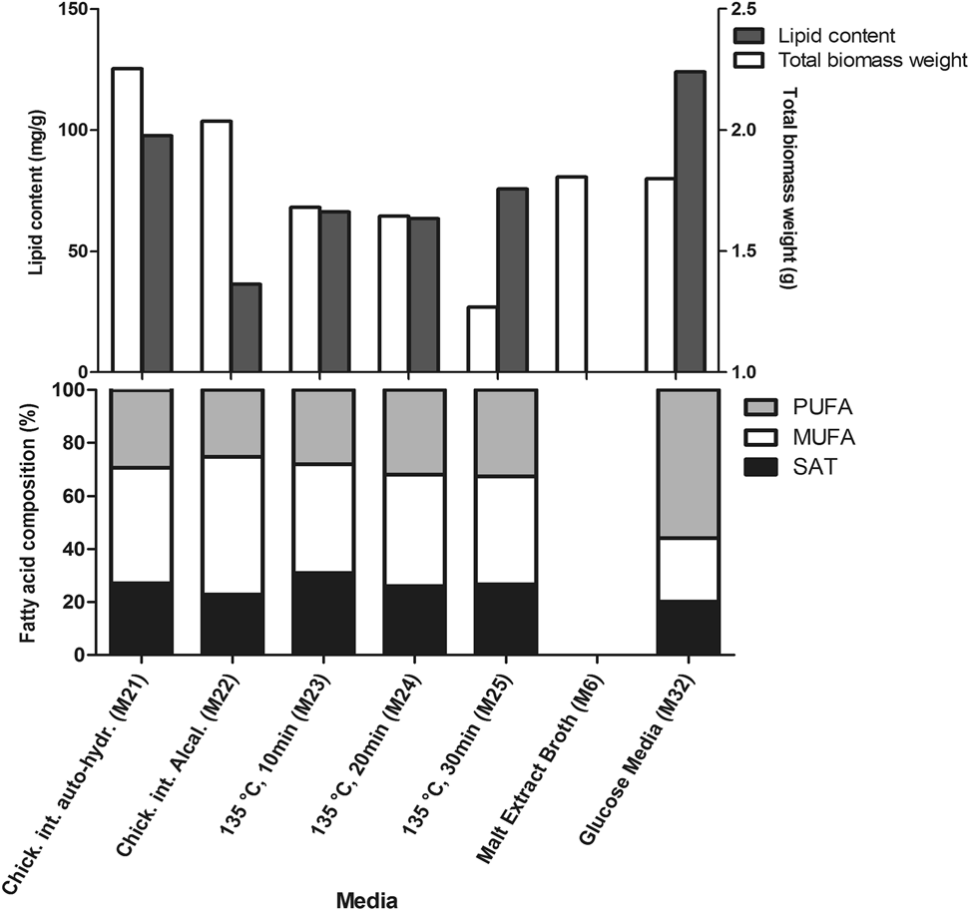
The total biomass weight, total lipid content and fatty acid profile of *M. circinelloides* grown on differently pre-processed chicken intestines-based media (M21–M25). The total biomass weight is expressed in g, the total fat content in mg/g of biomass and the fatty acid profiles (SAT, MUFA and PUFA) in percentages (%) of the total lipid content

All media based on the steam exploded and hydrolyzed chicken intestines (M21–M25) supported the growth of fungi and lipid production (Fig. 4). In general, the total biomass weight for the media based on the steam exploded chicken rest materials was lower than for control growth medium (M6). The media containing steam exploded chicken intestines from different combinations of temperature and exposure time (M23–M25) showed a lower total biomass weight when chicken materials steam were exploded at longer exposure time (M25). The total biomass weight for the media based on the hydrolyzed chicken rest materials were much higher than media containing steam exploded chicken intestines and control growth medium (M6), where medium with autolytic hydrolysates provided the highest total biomass weight (M21) (Fig. 4). Interestingly, the medium based on the chicken rest materials steam exploded at the longer exposure time (M25) resulted in higher total lipid content in comparison to the other steam exploded chicken intestine media (Fig. 4). The fatty acid profile of lipids from biomass grown on the steam exploded and hydrolyzed chicken rest materials was similar, and MUFAs were the dominating fatty acids (Fig. 4).

Based on above observed results, potato hydrolysate and chicken auto-hydrolysate were selected for the validation, where media evaluations were performed according to C/N ratios.

### Evaluation of media based on C/N ratios

The results presented in Table 6 show that media based on potato hydrolysate combined with chicken intestine auto-hydrolysates at two different C/N ratios could substitute glucose-based media of the same C/N ratios, producing comparable amounts of total fat, and a higher biomass yield.

**Table 6:** The total biomass weight, total lipid content and fatty acid profile of *M. circinelloides* grown on different media based on potato hydrolysate and chicken auto-hydrolysate mixed at different C/N ratios

Media	C/N ratio	Total biomass weight (g)	Lipid content mg/g biomass ± STD	SAT, %	MUFA, %	PUFA, %
M26	20	2.89	41.66 ± 9.86	19.26	39.94	40.65
M27	8	3.71	40 ± 0	17.44	49.49	32.94
M28	20	3.17	49 ± 2.64	20.78	46.35	32.62
M29	8	3.63	44 ± 3.46	21.56	38.53	39.59
M30	20	0.72	41.33 ± 2.51	19.20	18.79	61.96
M31	8	1.25	37.66 ± 2.51	19.26	29.36	51.35
M6		0.79		24.22	32.48	43.04

The total biomass weight is expressed in g, the total fat content in mg/g of biomass including standard deviation (± STD) and the fatty acid profiles (SAT, MUFA and PUFA) in percentages of total lipid content (%). Glucose and malt extract media were used as control growth and lipid production media, respectively

More specifically, the potato hydrolysate—chicken auto-hydrolysate medium (M28) with C/N ratio of 20 resulted in higher total fat content in fungal biomass compared to media containing glucose and yeast extract (M30, M31) with C/N ratios of 20 and 8, respectively, (*p* < 0.05) and compared to medium M27 (based on potato hydrolysate and yeast extract). However, glucose-based media resulted in higher PUFA percentages compared to the food rest materials-based media. The main PUFA produced was GLA. The concentrations in the biomass grown in rest materials-based media were higher compared to the one in malt extract broth and glucose medium at C/N ratio 8.

## Discussion

A possibility to use different types of rest materials from food and agricultural industry (waste frying oil, deproteinized cheese whey wastes, olive mill wastewater, wastewater from confectionary industries, wastes of soft drinks industry, potato wastewater, tomato wastes) for single cell oil production has been demonstrated successfully by several researches [21–24]. According to the authors’ knowledge, the evaluation of potato and animal food rest materials for the production of single cell oil was performed for the first time in this study. In the study the main focus was placed on testing different pretreatment procedures (steam explosion, enzymatic an autolytic hydrolysis) for potato and animal food rest materials, and evaluating the performance of model oleaginous filamentous fungus *Mucor circinelloides* grown rest materials-based media in comparison with control media, with respect to growth, total biomass weight and lipid content.

The elemental analysis of the steam exploded food rest materials as expected showed that potato and potato peel contain high C/N ratio. The high C/N ratio of potato and potato peel is explained by the high amount of starch and other carbohydrates, and low amount of nitrogen. The low C/N ratio for pork pulp and chicken intestines is explained by the high content of proteins. It has been reported previously that the increased C/N ratio in media can increase the fat content in *M. circinelloides* biomass [25]. As the lipid production media for *M. circinelloides* require a high C/N ratio, the results from the elemental analysis give a theoretical evidence that potato and potato peel alone could be a suitable substrate for *M. circinelloides* lipid production. Pork pulp and chicken intestine can be used as a nitrogen source for growth, but for lipid production additional glucose or other carbohydrate-rich materials is needed. But, by mixing potato/potato peel with pork pulp or chicken intestines in a proper C/N ratio we could potentially replace synthetic C- and N-source. This theoretical evidence was to some extent confirmed in the initial screening step, where synthetic C- and N-sources were replaced with sources based on steam exploded food rest materials. They were used in various combinations with commercial media components (M1–M5). The highest biomass weight was observed on the chicken intestine based medium (M3). The potato and potato peel-based media (M1–M2) provided both a low biomass and low lipid content while the amount of PUFA was high, despite of the high C/N ratio of the media. The reason for this could be due to the presence of inhibitors of both growth and lipid accumulation in the steam exploded potato and potato peel. The relative high content of PUFA in the fungi grown on potato and potato peel media remains to be explained.

When steam exploded potato peel, potato and chicken rest materials were combined, a much lower total biomass weight and lipid content was observed, in comparison to both control media and to the media where the same rest materials were combined with commercial medium components. This observation could be explained by the high level of chemical complexity of media based solely on the rest materials. It can be argued that the fungal cells in the utilization of complex media use most of their energy for degrading these complex chemical components down to the simple monomers that can be easily transported into the cells. To facilitate a higher degree of conversion of media components into cell growth and lipid production by increasing the level of monomers (glucose, peptides), steam explosion and hydrolysis procedures were tested.

It has been shown that steam explosion is an efficient processing way for the homogenization and sterilization of food rest materials. The use of steam explosion for pretreatment of potato/potato peel and chicken rest materials resulted in degradation and solubilization of starch and protein, respectively. However, still only a small percentage of starch and glucose compared to the initial amount was water soluble after steam explosion of potato and potato peel. In addition, a lot of water was incorporated into the steam exploded samples. Also, the use of very high steam explosion temperatures caused considerable glucose losses in potato and potato peel samples due to caramelization. The use of enzymatic hydrolysis for potato peel and potato rest materials resulted in a much higher release of water-soluble starch and glucose content. The use of endogenous and commercial enzymes for chicken rest materials resulted in a higher protein release compared to steam explosion. Based on the fact, that the use of endogenous enzymes showed similar results as the use of commercial Alcalase, the autolytic hydrolysis is suggested to be the best and cheapest way to pretreat chicken rest materials to be used in the fermentation.

It was hypothesized that enzymatically pretreated food rest materials may provide higher biomass and lipid yield compared to the steam exploded ones. This was confirmed during tests with media based on steam exploded and hydrolyzed rest materials combined with commercial media components. Media based on the hydrolyzed potato (M11) and chicken rest materials (M21) showed higher total biomass weight compared to steam exploded rest materials-based media. This might be due to the presence of growth stimulators in addition to high level of glucose, soluble starch, and proteins, in the hydrolyzed rest materials. The lipid yield of the hydrolyzed potato (M11) and chicken (M21) rest materials media was higher as compared to steam exploded rest materials-based media, but still it was very low in comparison to the lipid production in control lipid production medium (M32). This might be related to the use of a non-optimal C/N ratio. Thus, enzymatically hydrolyzed potato and chicken rest materials were selected to be used further for C/N evaluation. During the C/N ratio evaluation, the same C/N ratios were used for rest materials and control media. It was observed, that total biomass weight and lipid content for hydrolyzed rest materials-based medium was comparable or higher than for control glucose–yeast extract medium, while content of PUFA was lower. High biomass and lipid results obtained after growth on rest materials medium could be due to the presence of other nutrients, while the relatively low PUFA content remains to be explained.

Based on the results discussed above, enzymatic hydrolysis is shown to be the most efficient pretreatment process of the food rest materials chosen in this study for fungal lipid production. However, a special focus need to be placed on the study of the C/N ratio to be able to obtain high total biomass weight and lipid yield, as well as high contents of PUFA.
